# Response to treatment with grapiprant as part of a standard multimodal regimen in young dogs with appendicular joint osteoarthritis associated pain

**DOI:** 10.3389/fvets.2024.1461628

**Published:** 2024-10-24

**Authors:** Masataka Enomoto, Jonathan Hash, Tracey Cole, Maria D. Porcel Sanchez, Andrea Thomson, Erin Perry, Savannah Aker, Aoi Nakanishi-Hester, Emily Haupt, Logan Opperman, Simon Roe, Nichola Archer Thompson, John F. Innes, Benedict Duncan Xavier Lascelles

**Affiliations:** ^1^Translational Research in Pain Program, Comparative Pain Research and Education Centre, Department of Clinical Sciences, College of Veterinary Medicine, North Carolina State University, Raleigh, NC, United States; ^2^Department of Statistics, North Carolina State University, Raleigh, NC, United States; ^3^Elanco Animal Health, Hook, United Kingdom; ^4^Movement Independent Veterinary Referrals, Cheshire, England, United Kingdom; ^5^Center for Translational Pain Research, Department of Anesthesiology, Duke University, Durham, NC, United States; ^6^Thurston Arthritis Center, University of North Carolina, Chapel Hill, NC, United States

**Keywords:** osteoarthritis, pain, dog, grapiprant, force plate, fish oil, exercise

## Abstract

**Introduction:**

The response to medical management of young dogs with osteoarthritis (OA) associated pain has not been evaluated. Using an open-label design, the effectiveness, over a 4-month period, of standardized management (grapiprant/fish oil/exercise) for treating OA pain in young dogs was evaluated.

**Methods:**

Included dogs were 9 months-4 years of age; ≥3.6 kg body weight; had ≥1 appendicular joint with radiographic OA and obvious joint pain; had a Liverpool Osteoarthritis in Dogs (LOAD) score of ≥5. The non-steroidal anti-inflammatory piprant (grapiprant) was given at the recommended dose daily, omega-3 fatty acid supplementation was initiated at 100 mg/kg and then increased to 200 mg/kg daily, and leash exercise was gradually increased to a target of 60 min daily. Client-reported outcome measures (CROMs) and force plate gait analysis were collected at baseline and monthly for 4 months. The index limb was defined as the most severely affected limb at baseline.

**Results:**

Forty-eight dogs were enrolled (mean ± SD age of 30.7 ± 10.7 months). Hips, elbows, and stifles were commonly affected. Medication and supplement compliance was excellent (≥95% of target administered), and treatments were well-tolerated. CROMs showed significant improvement over time and at each time point. Overall, peak vertical force (PVF) increased significantly (<0.001), and vertical impulse increased numerically. Increase in PVF from baseline was significant at all time points except 4-months.

**Discussion:**

This study demonstrates a clinically meaningful benefit of a multimodal treatment regimen over a 4-month period for young dogs (<4 years old) with OA-pain. Future work should determine if early, effective treatment is of long-term benefit.

## Introduction

Osteoarthritis (OA) is a degenerative joint disease, often resulting in chronic pain (1). OA associated pain adversely affects multiple dimensions such as gait, function, and sleep (2), and its management can be challenging due to complex relationships between peripheral disease, nervous system input and changes over time, and comorbidities (3, 4). However, management is likely to be less complex earlier in the course of the disease. Therefore, earlier, effective treatment of OA-pain may better control joint pain and the longer-term negative impacts of joint pain on multiple dimensions, although this concept has not been tested. In the treatment of canine OA pain, a multimodal approach has been recommended including pharmacological agents, dietary modulation, and exercise and rehabilitation therapy (5).

In dogs, OA is thought to be initiated primarily by developmental joint disease (1). Recently, our group found that 40% of young dogs between 8 months and 4 years old had radiographic OA (rOA) in one or more appendicular joints and 40–60% of those dogs had joint pain (≥ moderate or mild pain, respectively) in one or more of the rOA joints (6).

Despite the high prevalence of OA-pain in young dogs and potential benefits of early treatment, no studies have evaluated the response to multimodal OA treatment in young dogs with OA-pain. The multimodal OA-pain treatment regimen employed in this study consisted of a nonsteroidal anti-inflammatory drug (NSAID), a nutritional supplement (omega-3 fatty acid supplement), and modification of exercise. Grapiprant is registered for the treatment of OA pain and is a non-COX-inhibiting, piprant class NSAID with a good safety profile (7). Omega-3 supplements are considered to be associated with efficacy in canine OA pain (5, 8, 9). Regular, low-impact controlled exercise is recommended to support movement, muscle strength and to help control body weight (10). The aim of this study was to assess the effectiveness of this standardized management plan for treating the clinical signs of OA in young dogs using objective and subjective outcome measures.

## Materials and methods

### Study design

This study was an open label evaluation of the response to multimodal treatment over a 4-month period in young dogs (9 months to 4 years old) with clinical signs associated with OA. The *in vivo* portion of the study was performed between June 2020 and November 2022. NC State University Institutional Animal Care and Use Committee (IACUC) approved this study and all procedures (IACUC#19-604-O), and the study was approved by the Hospital Clinical Studies Review Board. All dog owners signed a written consent form following a detailed verbal explanation of the study protocol.

### Sample size calculation

In this study, the primary outcome measure was the Liverpool OsteoArthritis in Dogs (LOAD) owner assessment. Based on pilot data from clinical management of dogs with OA pain, we expected a decrease of 4.5 points in the LOAD scores and pre- and post-treatment standard deviations of 6.77 and 7.60 respectively, and these data indicated that 80% power would be achieved with a sample size of 40 dogs. These data were from dogs ~8 years old.

### Recruitment

The study aimed to recruit 50 young dogs with clinical signs associated with OA. Osteoarthritic young dogs with clinical signs associated with OA that were identified in the previous prevalence study (6) were invited to participate in the current study. Additionally, young dogs with lameness due to OA pain were recruited to the study by advertisements via NCSU websites, e-mails to local practices and NC State employees, local radio advertisements and via CVM social media (Twitter and Facebook). Recruitment proved difficult (likely mainly due to the changes induced by COVID), and so Visionaire^1^ was employed and recruitment successfully completed via a targeted Facebook campaign.

### Case selection

To be eligible for the study, dogs were required to be between 9 months and 4 years of age at the time of recruitment, and ≥ 3.6 kg body weight. Dogs were required to have clinical signs of OA-associated joint pain confirmed by gait evaluation, veterinary assessment, and radiographic evidence, be in general good health or have stable chronic conditions and able to complete the study in the opinion of the veterinarian. Health status was assessed by physical examination, medical history, and clinical pathology evaluations (complete blood count, serum biochemistry profile, and urinalysis including sediment examination). The recruited dogs were also required to have a LOAD score of ≥5 and at least one joint with radiographic evidence of OA and a pain score of ≥2 out of 4 (moderate pain).

Dogs that had clinically relevant abnormal clinical pathology findings, spinal orthopedic abnormalities, or neurologic abnormalities that affected gait were excluded. Other exclusion criteria were concomitant disorders that may have affected evaluations for the study, and other joint diseases (such as immune-mediated joint disease). Dogs that had had major surgery within 1 month, or cruciate ligament surgery within 3 months were excluded, as were dogs that had surgeries that could confound the evaluation of OA pain (acute inflammatory pain due to surgery). A required wash-out period was at least 3 weeks for NSAIDs or short-acting steroids, and 4 weeks for long-acting steroids.

### Brief description of the study timeline

Owners signed an owner consent form before any study activities and then dogs were screened to see if they met the inclusion or exclusion criteria. A brief description of study outline is shown in Supplementary file 1. Outcome measures were performed, and blood and urine were collected for clinical pathology evaluations. Following veterinary assessments (physical, orthopedic, neurological), dogs were sedated for radiographs of all joints. Veterinary assessments and outcome measures (owner questionnaires, gait analysis) were also performed every month for 4 months. Blood work and urinalysis were repeated at the end of the study visit.

### Orthopedic examination

Physical, orthopedic, and neurologic examinations were performed, and data were captured. During the orthopedic examination, every joint of each limb was examined by a veterinarian experienced in evaluating pain associated with OA in dogs (ME), and joints were graded for pain, crepitus, effusion, and thickening. The manus and pes were considered as one joint region for evaluation purposes. Other appendicular joints evaluated were carpus, elbow, shoulder, tarsus, stifle, and hip. Spinal column segments were examined and graded for pain. The axial skeleton was evaluated by dividing the spine into cervical, thoracic (T1-9), thoraco-lumbar (T10-L6), and lumbosacral regions. Scores for pain ranged from 0 to 4. Assessments for crepitus, effusion, thickening, and range of motion were recorded, but not used in analysis. Scores were recorded on the Joint Evaluation Scoring SystEm canine (11). At screening, the Canine OsteoArthritis Staging Tool (COAST) was used for staging the impact of OA on patients (12). Based on the published papers, the items considered as the risk factors for OA in this study were orthopedic disease without radiographic evidence of OA (e.g. hip subluxation), traumatic joint injury/surgery, certain breed, overweight (BCS≥7) (13, 14).

### Radiography

Radiographs were taken under sedation with a mu-opiate combined with alpha-2 adrenergic agonist, for example, hydromorphone 0.05–0.1 mg/kg/IV and dexmedetomidine 0.003–0.005 mg/kg/IV. However, the choice of drug and dose was adapted according to the dog’s health condition. Orthogonal views of all appendicular joints and lateral views of the spine were taken. To minimize ionizing radiation exposure, where appropriate, radiographs were centered on the midpoint of the limb or spinal segment to reduce the number of individual exposures used. A subjective overall severity score was assigned to each joint based on a numerical rating scale where 0 = no radiographic abnormalities identified and 10 = most severe radiographic OA, as described previously (15, 16). Radiographs were assessed using a DICOM viewer (Horos ver. 3.3.6) by a veterinarian experienced in evaluating canine OA (ME).

### Treatment

Grapiprant was administered orally every 24 h according to the approved dosing chart to achieve a target dose of 2 mg/kg (7). Owners were instructed to give the dose 1 h before a meal and at approximately the same time each day. Compliance with dosing was evaluated by reconciling the returned pills with what was dispensed. 100 mg/kg of omega-3 fatty acid (fish oil; Nature Made, Viva naturals, Nutrigold) was added to the diet for the first week and 200 mg/kg of omega-3 fatty acid from the second week to the end of the study. The exercise protocol suggested varied based on the starting point of leash exercise for each case. Owners were advised to gradually increase leash exercise to 30 min twice daily or the equivalent thereof (adding 5–10 min of exercise every week). If a dog received leash-walking exercise for 60 min daily at the screening visit, no change was applied.

### Outcome measures

#### Client reported outcome measures (CROMs)

CROMs were used as previously described. The Liverpool OsteoArthritis in Dogs (LOAD) and Canine Brief Pain Inventory (CBPI) have been shown to be valid measures of the impact of OA- pain in dogs (17–21). Sleep and Nighttime Restlessness Evaluation Score Questionnaire version 2.0 (SNoRE) was used to collect data regarding sleep quality (22). The CROMs were completed by the dog owner. For the LOAD, the sum of each item score was calculated. For CBPI [pain severity scores (PSS) and pain interference scores (PIS)] and the SNoRE, the average of each item was calculated. A reduction of ≥4 in LOAD scores was defined as “minimal clinically-important differences (MCIDs)” as suggested by previous studies (23, 24).

#### Ground reaction forces (GRFs) measurement using a force plate (FP)

Inclusion criteria were not optimized for collection of data using a force plate (FP); therefore, FP data were collected only if the dogs ‘fit’ the FP system (i.e., were of a size such that GRF data would be collected using the FPs). GRFs were collected using dual in series FPs (AMTI, Watertown, MA, USA) and custom software (Sharon software, Dewitt, MI, USA). Velocity and acceleration were measured by means of five photoelectric cells placed 0.5 m apart and coupled with a triggered timer system (25). The dogs were trotted across the FPs at a velocity of 1.7–2.1 m/s and acceleration of each dog was restricted to mean acceleration at baseline ±0.5 m/s. A trial from which data was retained for analysis consisted of a full forefoot strike on each FP without another foot being on the plate at the same time, followed by an ipsilateral hindfoot strike in the same fashion on each FP. Thus, data from all four limbs were obtained in a single pass. A single trained observer evaluated each foot strike and subsequent force profile and determined whether or not the trial should be retained. A single handler gaited all the dogs for each trial and timepoint. Five valid trials were collected for each dog at each timepoint. Peak vertical force (PVF) and vertical impulse (VI) were the GRFs extracted, and the means of the five trials at each visit were used for analysis. All forces were normalized to body weight and expressed as a percentage of bodyweight. In all dogs from which GRF data were collected, an index limb (the most severely affected) was identified based on clinical signs of joint pain, muscle atrophy and limb use (regardless of if multiple limbs were affected). The change from baseline in PVF and VI of the index limb was calculated for analysis.

### Adverse events (AEs)

The owners were asked to report any unusual events during the study period. An AE was defined as any observation, undesirable experience, or reaction in animals that was unfavorable and unintended and occurred after the initiation of treatment, whether or not considered to be related to any treatment. Blood work and urinalysis were repeated at the end of the study visit.

The list of hematology parameters evaluated were: white blood cells, red blood cells, hemoglobin, hematocrit, mean corpuscular volume, mean corpuscular hemoglobin, red blood cell distribution width, reticulocytes count and its percentage, mean platelet volume, plateletcrit, platelet count, segmented neutrophils, lymphocytes, atypical lymphocytes, monocytes, eosinophils, neutrophils, basophils, packed cell volume, and plasma protein. Biochemistry parameters evaluated were: glucose, blood urea nitrogen, creatinine, phosphorus, calcium, magnesium, total protein, albumin, globulin, albumin/globulin ratio, cholesterol, total bilirubin, alkaline phosphatase, alanine transaminase, aspartate aminotransferase, gamma-glutamyl transferase, creatine kinase, sodium, potassium, chloride, bicarbonate, anion gap, sodium/potassium ratio, osmolality, amylase, lipase, hemolysis, and lipemia. Urinalysis parameters evaluated were: dipstick (Ph, protein, glucose, ketone, bilirubin, blood), color, urine specific gravity, white blood cell, fat, and sediment.

### Statistical analysis

All statistical analyses were performed using R version 4.2.2 or JMP software (JMP pro 16; SAS), *α* = 0.05 as our cutoff for statistical significance. For the CROM and GRF data, linear mixed models were fit with scores/variables as responses and time as a covariate with a random intercept for each patient. Each score/variable was also compared to baseline at each timepoint via Wilcoxon signed rank test with a Bonferroni correction then applied within each timepoint. For clinical pathology evaluations, the fit model function was used to compare the results collected before and after the study. An adjustment was made for multiple comparisons for clinical pathology evaluations.

## Results

**Figure 1:**
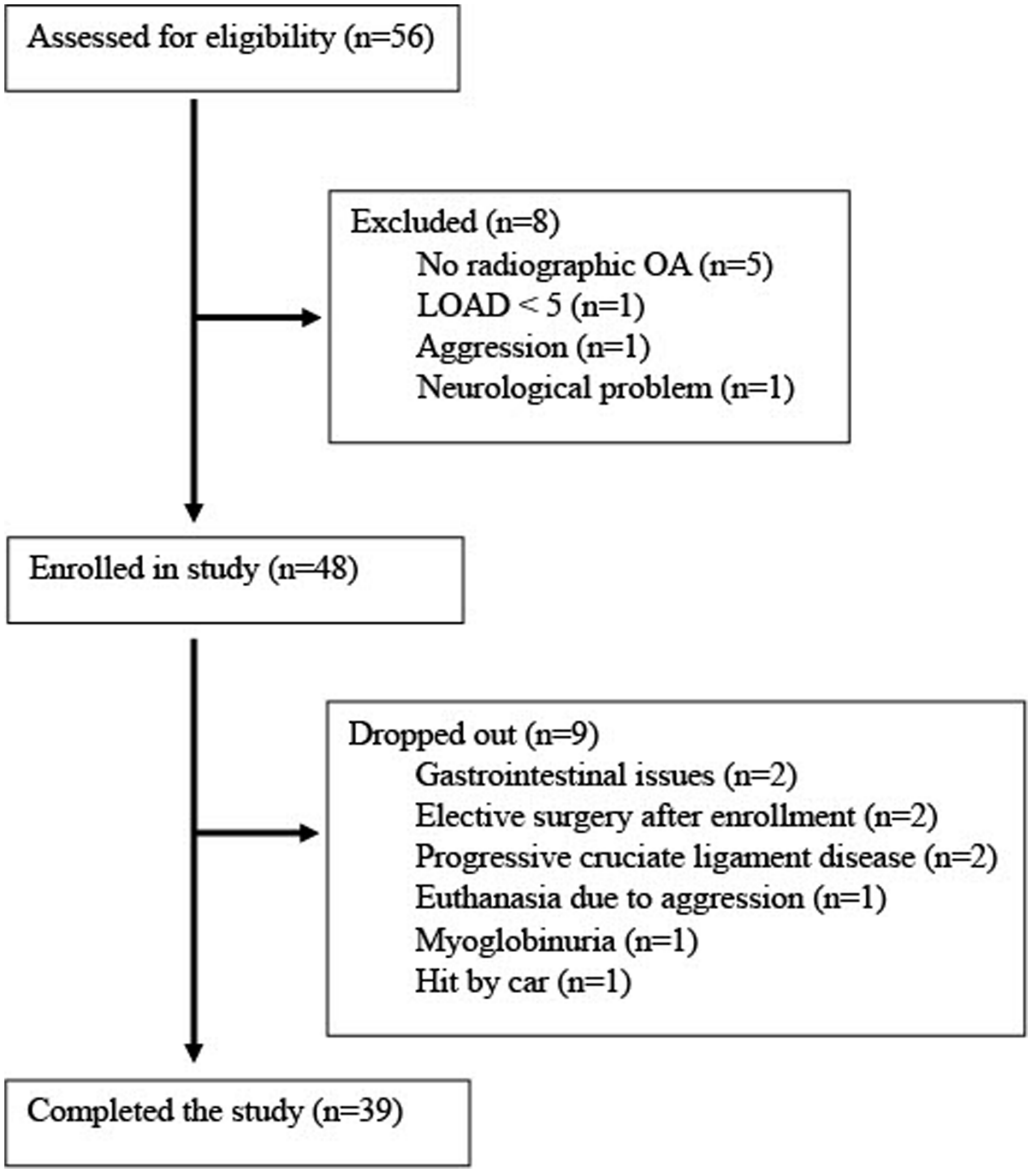
Flowchart of the clinical trial selection process showing the number of study patients at each selection process. OA, osteoarthritis; LOAD, Liverpool OsteoArthritis in Dogs.

Fifty-six dogs were assessed for eligibility for the study (Figure 1). Forty-eight dogs of 15 different breeds were enrolled into the study and 39 dogs completed the study. Nine dogs dropped out of the study prior to completion but efficacy data were included up to the time of dropout. One dog dropped out due to multiple GI issues, but the remainder were unrelated to treatment (elective surgery, progressive ligament disease, aggressive behavior, hit by car, myoglobinuria).

**Figure 2:**
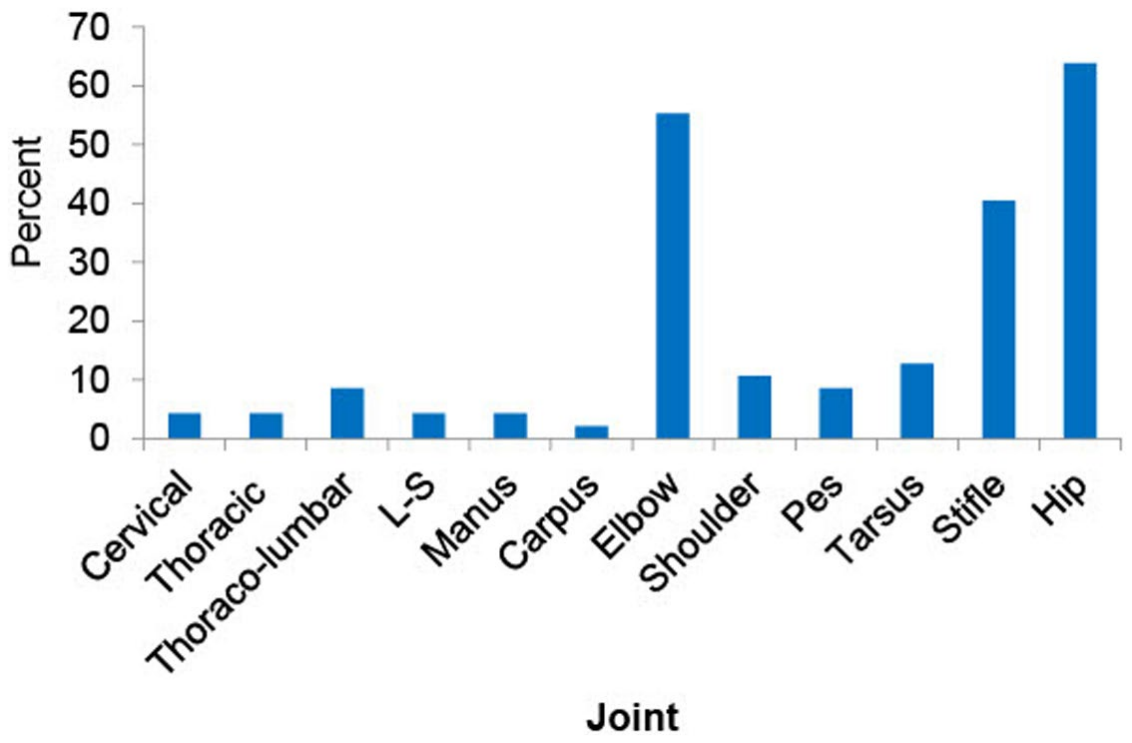
This figure shows the prevalence of radiographic osteoarthritis (rOA) across joints. The most commonly affected joints in order were hip, elbow, stifle and tarsus. L-S: lumbo-sacral joint.

Across all 48 dogs, mean (± SD) age, body weight, and body condition score were 30.7 ± 10.7 months, 30.5 ± 11.5 kg, and 5.4 ± 0.9, respectively. Six dogs were intact male, and 20 dogs were neutered male; 3 dogs were intact female, and 19 dogs were spayed female (Table 1). The most common breeds were mixed (*n* = 23), German Shepherd (*n* = 5), and Labrador Retriever (*n* = 5) (for full list of breeds see Supplementary file 2). Radiographically, the most commonly affected joints in order were hip, elbow, stifle and tarsus (Figure 2). Radiographic OA was present in one joint in 3 dogs, two joints in 15 dogs, 3–4 joints in 19 dogs, and ≥ 5 joints in 10 dogs (whole-body radiographs were not performed for one dog due to a heart problem). Mild or greater pain was detected in 71.5% of joints with rOA; pain was detected in one joint in 8 dogs, two joints in 22 dogs, 3–4 joints in 17 dogs. Nine dogs were recruited from the prevalence study (6) and 39 dogs were enrolled via study advertisement. Patient characteristics for these two groups are detailed in Table 2; LOAD scores, CBPI scores, and COAST stage were significantly lower in the dogs identified during the prevalence study than those identified via study advertisement.

**Table 1:** Mean ± SD (range) values of all the dogs enrolled in the study (*n* = 48).

	Mean ± SD (range)
Age (months)	30.7 ± 10.7 (11.0–51.0)
Sex	M: 6, F: 3, MC: 20, FS: 19
Body weight (kg)	30.5 ± 11.5 (9.0–68.9)
BCS (1–9)	5.4 ± 0.9 (4–8)
CBPI PSS	2.4 ± 1.9 (0–6.0)
CBPI PIS	2.6 ± 2.1 (0–7.6)
LOAD	16.1 ± 7.4 (5–33)
SNoRE	3.9 ± 1.3 (1.8–7.4)
COAST	3.3 ± 0.6 (2–4)

M, Male; F, Female; MC, Male castrated; FS, Female spayed; BCS, Body condition score; CBPI, Canine Brief Pain Index; PSS, Pain Severity Score; PIS, Pain Interference Score; LOAD, Liverpool OsteoArthritis in Dogs; SNoRE, Sleep and Nighttime Restlessness Evaluation Score; COAST, Canine OsteoArthritis Staging Tool.

**Table 2:** Mean ± SD (range) values of signalment and client-reported outcome measures in dogs transferred from the prevalence study and recruited specifically for this study.

	Prevalence study (*n* = 9)	Recruited for this study (*n* = 39)	*p*-value
Age (months)	32.4 ± 13.8 (14.0–45.0)	30.7 ± 10.0 (11.0–51.0)	0.66
Sex	M: 1, F: 0, MC: 6, FS: 2	M: 5, F: 3, MC: 14, FS: 17	0.30
Body weight (kg)	29.3 ± 7.5 (19.3–41.8)	30.8 ± 12.2 (9.0–68.9)	0.74
BCS (1–9)	5.4 ± 1.2 (4–7)	5.4 ± 0.8 (4–8)	0.86
CBPI PSS	1.1 ± 1.6 (0–4.8)	2.7 ± 1.8 (0–6.0)	**0.0145**
CBPI PIS	0.87 ± 1.4 (0–4.2)	3.0 ± 2.0 (0–7.6)	**0.0048**
LOAD	9.3 ± 4.8 (5–19)	17.6 ± 6.9 (5–33)	**0.0015**
SNoRE	3.9 ± 1.0 (2.6–5.4)	4.0 ± 1.4 (1.8–7.4)	0.93
COAST	2.9 ± 0.6 (2–4)	3.4 ± 0.6 (2–4)	**0.0172**
Radiographic OA score*	10.3 ± 7.1 (2.0–24.0)	10.7 ± 7.7 (1.0–29.0)	0.90
Number of joints affected	3.6 ± 2.0 (1.0–7.0)	3.3 ± 1.6 (1.0–8.0)	0.74

M, Male; F, Female; MC, Male castrated; FS, Female spayed; BCS, Body condition score; CBPI, Canine Brief Pain Index; PSS, Pain Severity Score; PIS, Pain Interference Score; LOAD, Liverpool OsteoArthritis in Dogs; SNoRE, Sleep and Nighttime Restlessness Evaluation Score; COAST, Canine OsteoArthritis Staging Tool.

*p* < 0.05 considered as significant (bolded).

*Whole body x-rays were not taken on 1 dog due to potential heart problem (this dog was removed from analysis), and the right hip joint was not scored due to femoral head and neck osteotomy/total hip replacement on 3 dogs (all recruited specifically for Part II: these dogs are still included in analysis).

Both overall and monthly compliance for medication/supplement were excellent. Overall, 97% of grapiprant/fish oil prescribed was utilized, and monthly compliance averaged ≥95%. The mean ± SD dose of fish oil was 91.7 ± 9.9 mg/kg for the first week and 196.0 ± 16.3 mg/kg from the second week. The average duration of exercise before treatment was approximately 40 min. Two dogs were reported to be unable to follow the exercise recommendation due to brachycephalic breed or progressive cruciate ligament rupture. The other owners reported they adhered to the exercise regimen, however, actual exercise undertaken was not recorded.

### Outcome measures

**Figure 3:**
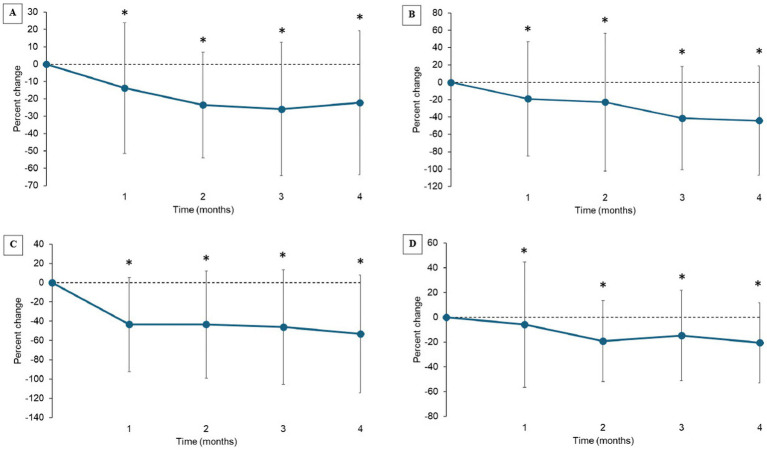
**(A–D)** Percent change from baseline in client-reported outcomes measures (Mean ± SD). **(A)** Liverpool OsteoArthritis in Dogs (LOAD); **(B)** Canine Brief Pain Inventory (CBPI) Pain Severity Score (PSS), **(C)** CBPI Pain Interference Score (PIS); **(D)** Sleep and Nighttime and Restlessness Evaluation Score (SNORE). *indicates significant difference from baseline (*p* < 0.05).

LOAD and SNoRE scores significantly (*p* < 0.001) improved over time. CBPI scores improved over time but did not fit the statistical model due to clustering around zero (Table 3). Scores each month were significantly improved compared to baseline for all the CROMs (Figures 3A–D). Using MCID values for LOAD, MCIDs were achieved in 42.2, 53.7, 53.7, and 43.6% of the patients at 1 month, 2 months, 3 months, and 4 months after the treatment, respectively.

**Table 3:** Mean ± SD (range) values of client-reported outcome measures at each time point.

	Screening (*n* = 48)	Post-1M (*n* = 44)	Post-2M (*n* = 40)	Post-3M (*n* = 40)	Post-4M (*n* = 39)	*p*-value
LOAD (0–53)	16.1 ± 7.3 (5–33)	13.3 ± 7.3 (3–32)	12.2 ± 6.4 (2–24)	11.6 ± 7.3 (1–26)	11.9 ± 8.0 (2–31)	**< 0.001**
CBPI PSS (0–10)	2.4 ± 1.9 (0–6.0)	1.9 ± 1.8 (0–6.0)	1.6 ± 1.6 (0–6.5)	1.4 ± 1.6 (0–5.3)	1.4 ± 1.8 (0–6.5)	Not fit
CBPI PIS (0–10)	2.6 ± 2.1 (0–7.6)	1.8 ± 2.0 (0–7.0)	1.5 ± 1.5 (0–5.0)	1.3 ± 1.7 (0–6.7)	1.3 ± 1.8 (0–7.1)	Not fit
SNoRE (0–10)	3.9 ± 1.3 (1.8–7.4)	3.5 ± 1.5 (1.2–9.0)	3.1 ± 1.3 (1.4–6.2)	3.3 ± 1.6 (1.2–7.8)	3.0 ± 1.5 (1.2–6.6)	**< 0.001**

CBPI, Canine Brief Pain Index; PSS, Pain Severity Score; PIS, Pain Interference Score; LOAD, Liverpool OsteoArthritis in Dogs; SNoRE, Sleep and Nighttime Restlessness Evaluation Score; M, month. *p* < 0.05 considered as significant (bolded).

**Figure 4:**
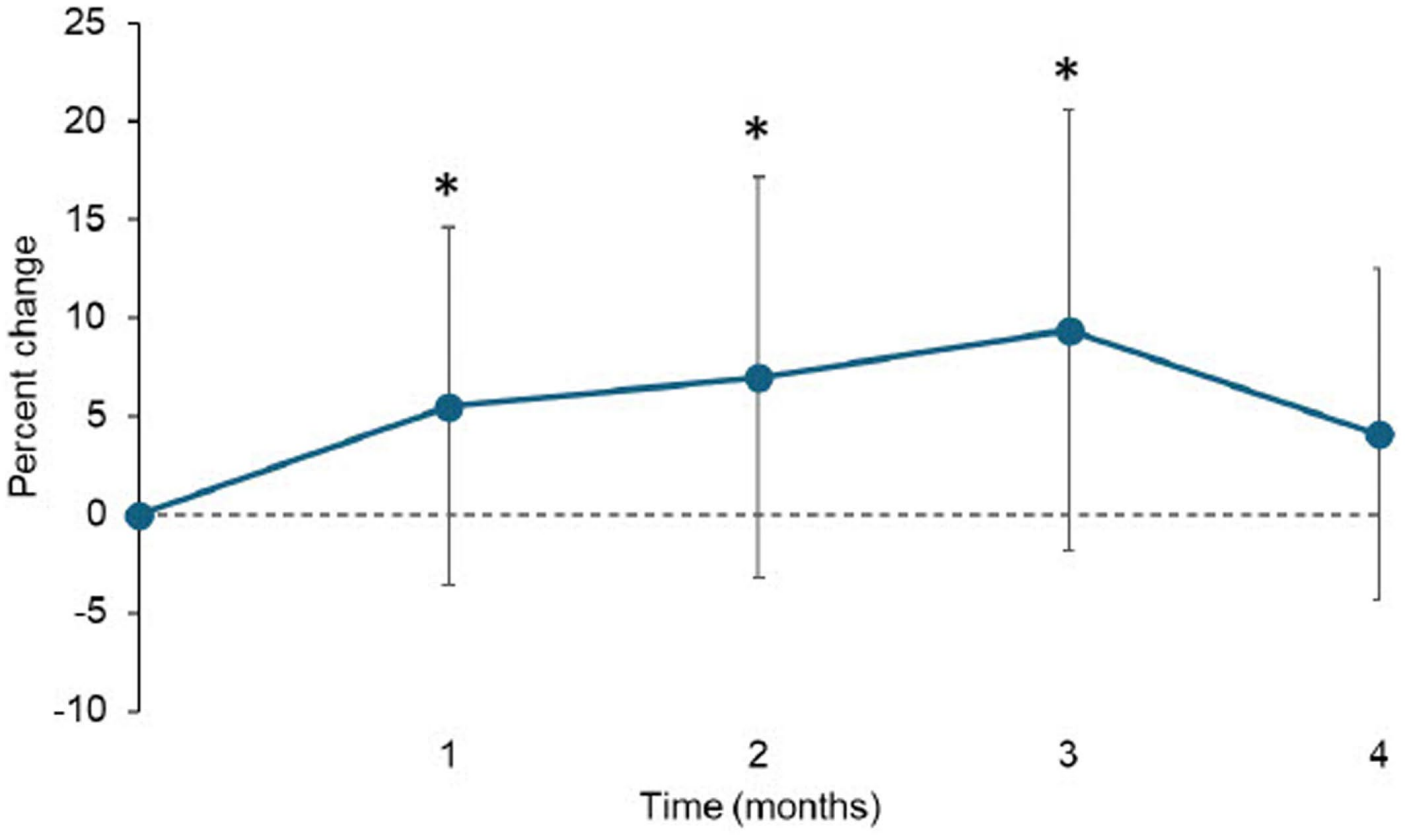
Percent change from baseline in the peak vertical force of the most affected limb (Mean ± SD). *indicates significant difference from baseline (*p* < 0.05).

The veterinarian-assessed joint pain score of the index limb significantly decreased over time overall (*p* = 0.046) but the change from baseline did not reach significance at any time point after correcting for multiple comparisons (Supplementary file 3). In twenty-five dogs GRF data could be collected. There was a significant increase in the peak vertical force (PVF, <0.001), and a numerical increase (not significant) in vertical impulse (VI, *p* = 0.209) over time (Table 4). The PVF increased an average of 1.02 percentage points per time point. The increase in PVF was significant at all time points compared to baseline except at 4-months (Figure 4; Supplementary file 4).

**Table 4:** Mean ± SD (range) values of gait variables at each time point.

	Time point				
	Screening (*n* = 25)	Post-1M (*n* = 24)	Post-2M (*n* = 24)	Post-3M (*n* = 25)	Post-4M (*n* = 23)
PVF (%BW)	77.3 ± 20.2 (45.9–121.8)	80.3 ± 19.8 (38.1–126.2)	81.8 ± 18.3 (54.1–122.2)	83.1 ± 19.5 (52.4–125.1)	79.6 ± 19.1 (47.8–5.3)
VI (%BW)	11.4 ± 3.9 (4.9–18.1)	11.5 ± 3.8 (4.6–18.4)	11.7 ± 3.6 (6.5–17.9)	12.0 ± 4.0 (6.1–19.0)	11.2 ± 3.7 (5.4–18.3)

PVF, Peak vertical force; VI, Vertical impulse; M, month.

### Adverse events

Although not all incidences were considered related to the treatment given, gastrointestinal AEs were in line with expectations for NSAIDs and fish oil use (vomiting, *n* = 7; diarrhea, *n* = 3; hyporexia, *n* = 2). The reported vomiting was, for the most part, a single occurrence, and classified as mild. In one dog, vomiting was classified as moderate for multiple episodes of vomiting a day for several days. This event was likely to be associated with the treatments given and the dog was withdrawn from the study. Another dog vomited a red color liquid several times just before the 2 months recheck, however, the dog was reported to have been chewing on a red color ink pen the day before the visit. Due to this reason, the association with the treatment was concluded as unlikely. However, this dog was withdrawn from the study. In one dog vomiting was treated by the regular veterinarian with maropitant (3 doses) and metronidazole (10 doses). All reported diarrhea instances were single occurrences and classified as mild. One dog was treated with probiotics and fiber supplements (5 days), but other cases resolved without treatment.

Other AEs are listed in Figure 1 and were considered unlikely to be related to the treatment given. The mean and median clinical pathology evaluations for screening visit and the end of study visit were within reference ranges. A statistically significant difference was identified between those visits for glucose (decreased), blood urea nitrogen (increased), creatinine (increased), and phosphate (decreased) when adjusting for multiple comparisons. However, only 1 dog was outside the normal range for each of these parameters (see Supplementary file 5), and no dog had values outside of the reference range in ≥ two of the tests above. No significant differences in any of the other parameters evaluated. Clinically meaningful changes were not seen in any dogs except for the dog who had myoglobinuria at its 1 month recheck. However, myoglobinuria was thought to be associated with a prolonged bout of unusually vigorous play with other dogs and it was reported that a similar episode had occurred prior to this study.

## Discussion

In this study, we evaluated the effectiveness of grapiprant as part of a standardized management plan for OA pain in young dogs using subjective owner assessments and objective gait analysis. The results showed that owner-assessed OA-associated clinical signs and objectively measured limb-use were significantly improved over a 4-month period in young dogs with OA-pain undergoing a standardized treatment regimen. This study showed that in an ‘open label’ context (similar to the situation in clinical practice) the combined treatment regimen appears to be effective and well-tolerated as a standardized multimodal management plan for treatment of OA-associated clinical signs and disability in young dogs.

The joint pain score of the index joint significantly decreased over 4 months of the study period following the treatment. Our assumption is that this was due to treatment. It must be remembered however that none of the joint pain scoring systems, including ours, have been sufficiently validated (5) and this was an open-label study. Ideally, a validated assessment tool should be used to conclude a treatment effect, and a placebo comparator group should be included. In interpreting our results, we are making the assumption that the joint pain score would have stayed the same had treatment not been instituted. In young dogs, the authors’ clinical experience suggests that a period of improvement in clinical signs and a reduction in assessed joint pain, can be associated with joint disease progression from acute to chronic; hip dysplasia is a prime example of this. However, this clinical experience has not been carefully documented. In this study, the dogs were of various ages and different clinical histories and it is unlikely that they were all enrolled at the precise time of acute to chronic transition.

CBPI, LOAD, and SNoRE have been validated as subjective measures to assess pain and/or clinical signs associated with OA in dogs (17–21). To evaluate the efficacy of new analgesics, two analytical methods have been commonly used; reduction of scores (pain; disability) from baseline and binary outcomes (success failure) (26, 27). In this study, based on CROM data, overall, pain and associated clinical signs were significantly improved with treatment. In published placebo-controlled studies investigating the efficacy of NSAIDs in dogs, a 20–40% reduction in LOAD or CBPI scores has been documented with treatment (19, 27). Although this study was an open-label study and many dogs enrolled in this study were only mildly affected, a similar degree of improvement was observed.

Binary outcome (e.g., success/failure designation) thresholds have been suggested for CBPI and LOAD changes over time (23, 24, 26). For CBPI, treatment success and failure have been defined, with success defined as a reduction of ≥1 in PSS and ≥ 2 in PIS from baseline (26). However, these criteria were made based on older dogs who were more impaired, so it is unknown how relevant these cut-offs for success/failure are in this young dog population. Furthermore, the starting point of CBPI was quite low in our study, and thus this approach was not applied to our data. However, as shown above, the percent change from baseline in CBPI PSS and PIS were statistically significant (Figures 3A–D). More recently, a reduction of ≥4 in LOAD scores from baseline was suggested as the MCID (23, 24). When this approach was applied to our data, approximately half of the dogs reached MCIDs at each time point following our standardized management plan.

One of the limitations of this study is that the CROMs used (e.g., LOAD, CBPI) were developed using older dog populations to quantify the severity and impact of chronic pain in dogs with OA, but were not designed to detect subtle and early signs of dog mobility issues; the LOAD was developed using dogs of mean ages 7.9 years and the CBPI was developed using dogs >5 years of age. Recently, the GenPup-M, a novel CROM, was published and it was suggested that it may be able to identify early mobility changes in dogs (28). However, this instrument was not available when this study was performed, and it has not been tested in a young dog population.

Gait analysis has been validated as an objective measure of changes in limb use as it relates to joint pain in dogs (27, 29, 30). In particular, peak vertical force (PVF) and vertical impulse (VI) have been used to determine efficacy of therapeutics in OA studies. A recent review paper suggested that a change from baseline in both PVF and VI over a short time period (less than 6 months) of 3.5% of baseline values (not %body weight (BW) change) is the minimum value that should be considered clinically important (29). In this current study, using force plate data, the change from baseline in PVF was an increase of 4.1–9.4% of the baseline values (Figure 4). In one study, change from baseline in PVF and VI was reported after 2 weeks of carprofen in dogs that appeared to be demographically similar to this current young dog cohort except for age. Although the data collection time point was different, the results were similar to the current study; the mean change in PVF was 3.2%BW and VI was 0.32%BW in that study and in the current study, the mean change in PVF and VI varied over the 4 months between 2.8 and 5.8%BW and 0.09 and 0.54%BW, respectively. The increase in PVF from baseline was not significant at the 4-month time point. There are several potential reasons for this; firstly, this may be due to natural fluctuation of this limb use measure. Secondly, and importantly, in this study, dogs were not enrolled based on an obvious single limb lameness (the majority of dogs had two or more joints affected) nor enrolled based on the ability to collect GRF data on our equipment. Gait analysis is an ideal outcome measure if a dog has lameness in a single limb. If multiple limbs are affected, dogs usually have complex gait abnormalities, and it is more challenging to interpret gait data especially when a systemic intervention is used because the intervention should affect all painful sites. Therefore, limb use changes are likely to occur across all limbs, and it may have been that the ‘index’ limb benefited most initially, but then other areas benefited, reflected in an apparent decrease in improvement in the index limb. Overall, an important fact to remember is that the percentage change from baseline in PVF of the index limb was above suggested meaningful change throughout the study period. Overall, the GRF data from the current study supports the conclusion that the standardized OA management plan improved limb use in young dogs. It is also possible that following an improvement in dogs’ mobility and ability to perform activities, changes in management of the dogs, including other types of exercise that were instigated, may have played a role.

There was a significant difference in LOAD, CBPI, and COAST between the dogs invited from the prevalence study and dogs recruited specifically for this study. Further analysis of COAST data revealed that the difference in COAST scores between these two cohorts of dogs was driven by the owners’ assessment. The dogs in the prevalence study were randomly selected from a database and their owners asked to visit the hospital for their dogs to receive a “health screen” (did not know their dogs’ joint health) while the owners of the dogs recruited for this study knew their dogs’ joint health (confirmed/suspected) and their dogs had a mobility issue. This may highlight that awareness of joint health status affects CROMs scores significantly, which was recently reported in cats (31).

From the perspective of being able to prove efficacy of the treatment regimen tested, the major limitation of this study is the lack of a placebo-group. Generally, a study needs to have a matched placebo treated group to be able to make strong conclusions about the efficacy of a treatment. This study was designed to look at the adherence to, and acceptance of, a standardized multimodal management plan for treatment of OA pain in young dogs and generate initial data on whether young dogs with OA-pain appear to respond to the treatment.

This open-label, pilot study demonstrates that young dogs (≤4 years old) derive a clinically meaningful benefit from a standardized multimodal management of OA-pain over a 4-month period. Future work should replicate these findings and confirm efficacy over placebo, and determine if such early, effective treatment is of long-term benefit.

## Data Availability

The raw data supporting the conclusions of this article will be made available by the authors, without undue reservation.

## Footnotes

^1^https://vrande.com
