# Supplementary material for: Response to treatment with grapiprant as part of a standard multimodal regimen in young dogs with appendicular joint osteoarthritis associated pain
Source: Front Vet Sci. 2024 Oct 24;11:1461628. doi: 10.3389/fvets.2024.1461628 (PMC11541952; doi:10.3389/fvets.2024.1461628)
Supplement: Supplementary file 1 [file Table_1.docx]

**Supplemental file 1.**

Brief study timeline. ‘X’ means the relevant activity was performed.

| Time point | Veterinary assessments | CROMs  Gait analysis | Clinical pathology | Radiography |
| --- | --- | --- | --- | --- |
| Screening | X | X | X | X |
| 1 month | X | X |  |  |
| 2 months | X | X |  |  |
| 3 months | X | X |  |  |
| 4 months | X | X | X |  |

CROMs: Client-reported outcome measures

**Supplemental file 2.**

List of breeds participated in the study (15 different breeds in total across 48 dogs)

Mixed breed: 23 dogs

Mixed: 13

Pitbull mix: 3

Labrador retriever mix: 2

American Staffordshire terrier mix: 1

Rottweiler mix: 1

German Shepherd mix: 1

Spaniel mix: 1

Border Collie mix: 1

German Shepherd: 5 dogs

Labrador Retriever: 5 dogs

Golden Retriever: 3 dogs

Australian Shepherd: 2 dogs

Australian Cattle dog: 1 dog

Staffordshire terrier: 1 dog

English bulldog: 1 dog

Bullmastiff: 1 dog

Vizsla: 1 dog

Great Pyrenees: 1 dog

Great Dane: 1 dog

Basset Hound: 1 dog

Rottweiler: 1 dog

Border Collie: 1 dog

**Supplemental file 3**.

Mean ± SD (range) of the joint pain score of the index joint at each time point.

| Time point | Mean ± SD (range) |
| --- | --- |
| Screening | 2.43 ± 0.54 (2-4) |
| 1 month | 2.18 ± 0.77 (0-4) |
| 2 months | 2.17 ± 0.77 (1-4) |
| 3 months | 2.31 ± 0.91 (0-4) |
| 4 months | 1.97 ± 0.84 (0-4) |

**Supplemental file 4.**

Mean ± SD (range) values of percent change from baseline in gait variables at each time point

|  | Time point | | | |
| --- | --- | --- | --- | --- |
|  | Post-1M (n=24) | Post-2M (n=24) | Post-3M (n=25) | Post-4M (n=23) |
| PVF (%) | 5.5 ± 9.1  (-17.3 – 25.7) | 7.0 ± 10.2  (-16.0 – 33.6) | 9.4 ± 11.2  (-10.5 – 37.6) | 4.1 ± 8.4  (-9.7 – 20.6) |
| VI (%) | 3.4 ± 11.1  (-9.3 – 35.1) | 4.5 ± 13.8  (-11.8 – 37.9) | 6.1 ± 9.9  (-12.7– 33.9) | 1.2 ± 8.9  (-14.8 – 23.0) |

PVF: Peak Vertical Force, VI: Vertical Impulse. M: month

**Supplemental file 5**.

Clinical pathology parameters with a significant change from baseline (mean ± SD, range. The number of cases going outside the reference range are included. Adjustment was made for multiple comparisons, critical p-values = 0.0027 (hematology) and 0.0018 (biochemistry) Parameters marked * are significant with corrections for multiple comparisons.

| Test  (normal range) | Baseline | The end of the study (increase; decrease) | P-value | # of dogs went outside of range (up; down) |
| --- | --- | --- | --- | --- |
| Atypical Lymph  (no reference) | 1.13 ± 1.0  (0.19 – 3.9) | 0.75 ± 0.58  (0.079 – 2.2) | **0.0498** |  |
| Hgb  (13.7-21.0) | 16.2 ± 1.1  (13.2 – 18.0) | 16.9 ± 1.5  (14.5 – 20.8) | **0.036** | 0 |
| Hct  (40.2-61.2) | 47.4 ± 3.4  (38.8 – 53.2) | 49.5 ± 4.5  (41.6 – 62.7) | **0.02** | 1 (53.1 to 62.7) |
| PCV  (39-58) | 45.7 ± 3.4  (37 – 52) | 47.8 ± 4.5  (40 – 61) | **0.02** | 1 (51 to 61) |
| Glu  (75-126) | 104.3 ± 14.4  (75 – 129) | 93.5 ± 8.6  (72 – 116) | **< 0.001*** | 1 (109 to 72) |
| BUN  (11-27) | 14.9 ± 3.0  (10 – 21) | 18.9 ± 3.7  (10 – 26) | **< 0.001*** | 1 (11 to 10) |
| Cre  (0.5-1.4) | 0.94 ± 0.14  (0.6 – 1.2) | 1.09 ± 0.21  (0.7 – 1.7) | **< 0.001*** | 1 (0.9 to 1.7) |
| Alb  (3.2-4.3) | 3.7 ± 0.22  (3.3 – 4.1) | 3.8 ± 0.27  (3.2 – 4.4) | **0.03** | 1 (4.1 to 4.4) |
| Phos  (2.6-5.3) | 4.7 ± 0.72  (3.0 – 6.2) | 3.9 ± 0.63  (2.9 – 5.4) | **< 0.001*** | 1 (5.4 to 5.4): no change |
| Mg  (1.9-2.5) | 2.0 ± 0.11  (1.7 – 2.2) | 2.0 ± 0.13  (1.7 – 2.3) | **0.01** | 1 (1.8 to 1.7) |
| K  (3.6-5.3) | 4.3 ± 0.30  (3.6 – 4.9) | 4.5 ± 0.40  (3.7 – 5.9) | **0.007** | 1 (4.2 to 5.9) |

Hgb: hemoglobin, Hct: hematocrit, PCV: packed cell volume, Glu: glucose, BUN: blood urea nitrogen, Cre: creatinine, Alb: albumin, Phos: phosphorus, Mg: magnesium, K: potassium
